# President’s Address1Pennsylvania State Veterinary Medical Association, Annual Meeting, Philadelphia, Pa., March 3 and 4, 1903.

**Published:** 1903-05

**Authors:** W. L. Rhoads

**Affiliations:** Lansdowne, Pa.


### THE JOURNAL

OF

### COMPARATIVE MEDICINE AND VETERINARY ARCHIVES.

Vol. XXIV. *MAY, 1903.* No. 5.

### PRESIDENT'S ADDRESS.[1]

W. L. Rhoads, D.V.S.,

LANSDOWNE, PA.

It is with sincere pleasure I extend to you the hand of good fellowship, which carries with it the appreciation of your presence at this time.

A profession to be most successful must be combined, that the greatest good may accrue to the individual member of that profession and the greater results accomplished through the systematic concentration of the professional efforts under association tutorage and guardianship.

The professional man of good moral character and social standing by complying with certain requirements may, as a rule, become a member of his local, State, or national association. While membership is helpful, this alone does not make him a factor in the advancement of his profession. The advancement of all professions is due to combined efforts as directed through the associations.

Never before has association progress and hustle borne the rich returns that it is to-day. The last annual meetings of the New Jersey, Iowa, Minnesota, and Pennsylvania Veterinary Medical Associations were by far the best in their history. The local associations are also holding meetings of unusual interest. A great amount of this interest is no doubt due to the secretaries, who have advance methods and the past experience of their predecessors to help and guide them. Their post is one of honor, payable only in a great responsibility and limitless work. It is

[1] Pennsylvania State Veterinary Medical Association, Annual Meeting, Philadelphia, Pa., March 3 and 4, 1903.

undoubtedly the secretary who does much toward keeping the association grist-mill full and running smoothly and grinding out a fine and impalpable education, as it were, that is absorbed by and is nourishing to all progressive veterinarians, and they are all association men. Yet as a past secretary I cannot but feel that some of the noticeable progress is due to an increased interest of our association members, who have at the request of their secretary furnished a programme of good and wholesome topics, thus making our meetings of unusual interest and importance; thus stimulating and encouraging every veterinarian to greater efforts in the discharge of his duties to his association and fellow veterinarians by the contribution of something toward this advancement.

Fellow-men: I have always been thankful that a kind Providence allowed me to take up my life's work in a State in which the personnel of my chosen profession was composed chiefly, if not entirely, of men in the fullest, truest interpretation of that word. True, there may be some of us who are not all that our fellow practitioners desire; we all have our shortcomings and differences; true, we do not always agree upon minor points. Yet, when it comes to questions of moment and importance, we are as one.

Zenobia said: "I am charged with pride and ambition; the charge is true and I glory in its truth." It is so with the veterinarians of Pennsylvania individually and collectively. Fellow veterinarians, we have much to be thankful for. We have yet to have one of our number to betray the trust imposed in him and for self-aggrandizement or personal gain turn traitor to those people and principles which he should hold sacred and true, thus causing the finger of scorn and derision to be pointed at him or his association and State throughout the Union.

You cannot know how I appreciate the honor of being one of you or how I appreciate the honor of your Presidency bestowed upon me, not in your younger and immature years, but after gaining your majority, after passing the twenty-first milestone, as it were, upon the pathway of your existence and experience—a pathway which each year has widened out with your increased membership; a pathway which is widening into a broad highway, and, I trust, an improved highway, which we hear so frequently of these last few months.

The veterinary profession of Pennsylvania may congratulate itself upon this unanimity of effort when seeking the greater and more essential things; to this factor more than any other is undoubtedly due our phenomenal growth.

We have asked only for legislation as a dire necessity and only after thorough discussions, and we have invariably received due consideration at the hands of our law-making bodies.

It is no doubt due to the unanimity of action that we owe the retention of our State Veterinarian throughout succeeding administrations—a retention the munificent benefits of which are becoming more apparent year by year—and I wish to take this opportunity to extend to you, gentlemen, my heartfelt thanks and appreciation for your prompt response to one who was selected of our number to bring to your attention—not the necessity, as we feel the present Governor is interested not in politics, but the general welfare of his people, but the propriety of requesting the appointment of Dr. Leonard Pearson as State Veterinarian, more that the Governor might know it was the wish of the people; not that we feared he would remove one whose true worth he already knew and appreciated, and I would here ask that a vote of thanks be extended to Governor Pennypacker for his prompt acquiescence of our request.

We of Pennsylvania readily realize the power of concerted action, though we are undoubtedly strong enough as a profession to wield a mighty influence if our powers are unanimously exerted. We have been taught to thoroughly realize that "in union there is strength," and through disconcerted action we lose all. We never at any time came so near completing a great work; a work which accomplished would have placed our national army equipment on a par with the best of the world, instead of leaving it as it is to-day, a laughing-stock for the entire world; an institution upon which our politicians can spend millions for junketing tours and self-aggrandizement. Yet without a veterinary army corps, an institution whose very heart in the time of need will be found to be eaten by the canker worm of jealousy, for all depend in active warfare upon rapid mobilization, which cannot be accomplished without a thoroughly good equine equipment to be had to the best advantage only through the organized efforts of an organized army corps. An organization that would have been an honor to our nation as well as a money saver, and thus a good investment; an organization that would not only have paid for itself to the nation, but in its saving from what is now a woful, wilful waste, would have returned to our nation a handsome dividend. An organization that would have done much for the horse owner, breeder and raiser; an organization that would have been an honor to our profession. An organization which owed its conception and accomplishment to one whom the politician and his kindred spirit could not lead astray; one who devoted his life to the advancement of the profession; one whose life professionally might be emulated by every veterinarian with personal gain and much credit thus to accrue to the profession. I speak of one who is no more among us; one whose loss those who knew him best most deeply feel, namely, Rush Shippen Huidekoper. His endeavor to complete a veterinary corps—the most beneficial movement in the general advancement of our army known to history—as the crowning of his life's work, was frustrated by a small, disagreeable element led by a Judas Iscariot, who, through his willingness to do the bidding of men who have not the courage of their convictions, has attained to official positions of a sister organization of a neighboring State.

The future of the veterinary profession is at the present time lined with a more roseate hue than ever before. The increase of horse valuations within the last three years has been phenomenal and will undoubtedly continue, being due to the inadequacy of the supply as compared with the demand, and this deficiency is going to increase year by year, until we have the practicably safe flying machine, it being the only mechanical device to even partially supplant our equine friend.

The advent of the bicycle, the trolley car, and automobile, which so many hailed with delight and prophesied that each of them in turn sounded the death knell of the horse, did naught but increase our appreciation of the true worth of the noble steed. The bicycle we seldom see, excepting as a child's method of amusement, or in the hands of the laboring man, who, having a good position away from home, is pushing it along. The automobile is as yet a toy, useful, it is true, in the hands of the wealthy; yet a toy which is destined within a few years to descend from its pedestal of sport and racing qualifications to that of our heaviest hauling and drudgery; if not, then it shall pass with history down the annals of time as an inglorious failure. In spite of all the machinery invented to supplant the horse, he not only holds his own, but will year by year increase his prestige.

With the formation of road drivers' associations throughout our land we will ultimately have the horsemen of this country banded together in one great, fraternal body, the chief aim of which shall be the improvement and advancement of the horse, and to the veterinarians more than to any other body of men should this project appeal. Individually and collectively they should put their shoulders to the wheel and work for the common good of the masses. To quote from one of our periodicals: "Society owes to the horse a debt of gratitude a thousand times greater than it does to thousands of men who abuse him. He has ministered to progress; has made social intercourse possible when otherwise it would have been slow and occasional, or altogether impossible. He has virtually extended man's possibilities by increasing his strength, augmenting his speed, doubling his time, decreasing his burdens, and becoming his slave; has relieved him from drudgery and made him free." For love's sake, for the sake of social life, for eminent moral reasons, the horse deserves to be bred, trained, cared for with scrupulous care; and how can we better prove to him our appreciation of the horse of the past, the horse of the present, and the horse of the future, than by giving him improved highways to travel upon, that we may thus lessen his distance and lighten his load? How can this work better be accomplished than by a banding together of kindred spirits from each community, the most actively interested man of whom should be the local veterinarian, as his life-work is a benefaction to not only the horse but all other domesticated animals? Go into it for the general good; not from the business point of view, though you may be so judged. Remember, the man who goes to church and drops a nickel on the plate may feel that it is all it is worth to him; being so used to riding in the trolley car, he expects to reach heaven at the same rate of fare, while the fellow who puts in a dollar may feel he is far from the pearly gates, and fearing the long summer season awaiting him, contributes generously that the journey may be made more comfortable.

Fellow veterinarians, regardless of the judgment of the narrow-minded few, go in and hustle for all that is good and wholesome for your community, your county, and State. Another important factor to the welfare of the veterinarians is the appreciation by your people of the dire necessity of an improved milk supply, coupled with their consideration of the improved milk as a healthy, wholesome food, thus causing the demand to exceed the supply, leading the farmer or dairyman to appreciate the worth of his milk or dairy cow; in fact, stronger prices throughout the field of his production cause him to have a greater appreciation of the veterinarian's skill. I cannot but feel we might be of far greater value to our clientele if we, with all our other virtues and shortcomings, combined that of dieteticians. With some study we might give much valuable advice upon not only the profitable feeding of farm animals, but as well upon the diet of the coming two-minute trotter or valuable roadster.

The recent graduate in veterinary medicine, given a full scientific equipment, with his heart and honor in his work, who has a thorough conception of the great field before him, who appreciates but a few of the scientific mysteries hidden therein that he may garner for the advancement of science and the edification not only of scientific men but for the benefit of the masses, may do more toward warding off and preventing dire disease through his thorough knowledge of municipal meat and milk inspection when it is left to his scientific judgment, and he be not subservient to the whim of some political rounder who is kept in power by a gang of boodle politicians, who sacrifice regardlessly the lives of the men and women who are forced to gain their daily livelihood within the confines of our larger cities; who are forced to subsist upon an unwholesome food supply, inspected probably at best, if it is inspected at all, by an incompetent political inspector, thus making Ruffs and White Haven Institutes necessary throughout the land.

Stop and think of the great mortality among the little children of New York, Chicago, Pittsburg, Baltimore, and Philadelphia, annual victims of an impure milk supply. I will guarantee there is enough filth served to the people of Philadelphia in their supposedly pure milk each year to convert the most arid one-hundred-acre farm in this commonwealth, if used thereon as a fertilizer, far more fertile and productive than the land of Goshen dare be in the height of its prosperity. We cannot expect a great improvement unless the consumer will look more to health and less to the almighty dollar. We hear the cry of charity at all times and see the walls of charitable institutions reared through philanthropy, and by people who endeavor to do for the poor unfortunates to the best of their knowledge gained by years of experience. Yet a canvass of these institutions, including hospitals, where every care and precaution should be taken, finds them without exception buying in the open market, their chief aim being the lowest price. When it comes to a question of providential interference from a standpoint as regards our municipal meat and milk inspection, or political non-interference so long as the perquisites are right, politics seems to outtop Providence every time.

What are we going to do about it?

With a good, energetic press committee in every local organization that is in the least interested in the health and welfare of that community, the masses can be awakened to their danger and this yearly sacrifice prevented.

I wish also to speak of two branches of veterinary science that in the past have been invaded by empirics, namely, castration and veterinary dentistry. The technique of each should be placed upon a scientific basis, and we should endeavor to inculcate into the mind of the owner that it may be done to an advantage if done by one who is thoroughly conversant with the technique of his subject. But first be sure you have the matter well in hand; do not allow some empiric to run you into a corner with unanswerable questions.

We do not wish to overlook the question of milk examination. While it is not necessary for the veterinarian to be an expert chemist, this is a subject directly in line with his work, and he should be the first man consulted, and in return should be able to give some definite answer to constituents and condition. He should know what diseases are liable to be carried and disseminated through this channel, and how, as well as when and where, they would be most likely to be found.

Another matter of importance to the veterinary profession is the criticisms and ridicule, editorial and otherwise, indulged in quite frequently by our daily papers and periodicals. I feel this is a subject which should receive vigorous handling at the hands of our Press Committee, who will undoubtedly not only advocate all that is helpful to the profession, but are ready to defend it at every turn through the columns of the daily press, if the individual members will bring these matters to their attention.

I would suggest that in the near future some individual member or a committee take up the matter of new remedies or the improved combination of remedies, new or improved instruments, etc.; in fact, any new item which may be of interest to the profession individually or collectively, as we can only hope to obtain the confidential support of the public by proving to them that we are in thorough accord and sympathy with the modern march of advancement, and it is for a furtherance of this object that we to-day have the representatives of the leading drug and instrument houses of the country with us.

Our last semi-annual meeting was held at Reading under most favorable auspices and surroundings, and was undoubtedly one of the most successful and prolific semi-annual meetings within the history of the Association—due to a great extent to the untiring, unflagging interest of our worthy Secretary that this semiannual meeting was a success, and we probably appreciated it from the fact that the first semi-annual meeting of the Association was held in that town during September, 1883.

Last September I spoke of the needs and benefits of more local associations, also the benefits of a black list as compiled by the various associations. I suggested that the county secretaries take the matter up and form local protective associations in every county, then we may have monthly or semi-monthly reports throughout the State. The compilation and dissemination of this work through our Press Committee means untold advantages to accrue to our profession. Though suggested six months ago, I fail to see that it has been taken up; at least it has not brought results with alarming rapidity. I would again press upon you the urgent need of such a movement, and the county secretaries are the men who should take it in hand; they should also keep the chairman and other members of the Press Committee posted as to all matters of veterinary interest transpiring in their vicinity, that the Association and the profession may at all times be posted and thus guarded and profit thereby. The individual member should consider it his duty to assist not only his county secretary, but his association and association officers in every way at his command. Now, I do not feel this applies particularly to your brother veterinarians, for thou art the man.

Our position to-day is due to the pioneers who took up this glorious work at a time when there was no money, much labor, no honor, apparently but slight credit at any time to accrue to the calling in the future. We may look back with pride to the work of the Micheners, the Rayners, and our late lamented friend and coworker, Rush S. Huidekoper; their work has been followed by younger men, who have been probably better equipped for the work before them by the benefits of a later-day education. Men who have imbibed the same zeal, honesty, and integrity that ever characterized the lives of those men. Among our present workers I would only mention a few, viz., Kooker, Pearson, Hoskins, who have done much in the past, and are doing yeoman service at the present time. This burden must be shared and it must be taken up by a larger number of association workers, chief among whom should be the county secretaries.

The registration of our practitioners in Pennsylvania should be thoroughly considered at this time in the formation of all new laws; there are apt to be loopholes through which there are undesirable leakages in and out. This question of registration needs new legislation, and new registration should be asked for by all who are entitled to legitimate practive and the recovery of the fees commensurate therewith.

I would suggest that our coming Legislature take this matter up and make a new registration act, where registration must be made through our State examining board, thus obviating the chance for unqualified men to longer continue to menace the welfare of those who have already qualified through years of steady, active practice, or the medium of a standard veterinary college. In connection with the passage of this act, I would suggest a clause allowing the claim for professional fees to take priority of all excepting that of the final reaper.

This also brings to mind the urgent need of an act demanding the registration and licensing of all stallions kept for breeding purposes, in order that those animals which are to be progenitors of our future horse supply shall ensure to their offspring conditions free from the many unsoundnesses whose tendencies are transmissible from parent to offspring.

I wish to speak of the appreciable work of our State Board, of the new veterinary school to be erected at the University of Pennsylvania, of the proposed library there, of our loss through death of some valued member; yet these are matters which will come up in their regular order in the proper channels and have your hearing and consideration.

Having taken so much of your time, I will take this opportunity and extend to you my heartfelt thanks for your hearty co-operation and assistance, and my appreciation for your ready response to my most frequent appeals through a long period of secretaryship. I also feel deeply grateful to the individual members, to the committees, the county secretaries, and my brother officers for their hearty support through my term as your President.

Thanking you for the honor conferred upon me and asking that you may be as considerate of my successor in the future as you have been of me in the past, I can only say my most earnest wish is for a long, prosperous life for the individual veterinarians, for the local, State, and national organizations.

---

SERUM FOR PNEUMONIA. Professor Tizzoni, of the Bologna University, has announced to the Royal Academy of Sciences the discovery of a serum to combat pneumonia..

